# Longitudinal association of physical activity and sedentary behavior during leisure time with health-related quality of life in community-dwelling older adults

**DOI:** 10.1186/1477-7525-9-47

**Published:** 2011-06-27

**Authors:** Teresa Balboa-Castillo, Luz M León-Muñoz, Auxiliadora Graciani, Fernando Rodríguez-Artalejo, Pilar Guallar-Castillón

**Affiliations:** 1 Department of Preventive Medicine and Public Health. School of Medicine. Universidad Autónoma de Madrid/IdiPAZ-CIBER of Epidemiology and Public Health (CIBERESP). Madrid. Spain

## Abstract

**Background:**

Evidence on the relation between leisure-time physical activity (LTPA) and health-related quality of life (HRQoL) in older adults is based primarily on clinical trials of physical exercise programs in institutionalized persons and on cross-sectional studies of community-dwelling persons. Moreover, there is no evidence on whether leisure-time sedentary behavior (LTSB) is associated with HRQoL independently of LTPA. This study examined the longitudinal association between LTPA, LTSB, and HRQoL in older community-dwelling adults in Spain.

**Methods:**

Prospective cohort study of 1,097 persons aged 62 and over. In 2003 LTPA in MET-hr/week was measured with a validated questionnaire, and LTSB was estimated by the number of sitting hours per week. In 2009 HRQoL was measured with the SF-36 questionnaire. Analyses were done with linear regression and adjusted for the main confounders.

**Results:**

Compared with those who did no LTPA, subjects in the upper quartile of LTPA had better scores on the SF-36 scales of physical functioning (β 5.65; 95% confidence interval [CI] 1.32-9.98; p linear trend < 0.001), physical role (β 7.38; 95% CI 0.16-14.93; p linear trend < 0.001), bodily pain (β 6.92; 95% CI 1.86-11.98; p linear trend < 0.01), vitality (β 5.09; 95% CI 0.76-9.41; p linear trend < 0.004) social functioning (β 7.83; 95% CI 2.89-12.75; p linear trend < 0.001), emotional role (β 8.59; 95% CI 1.97-15.21; p linear trend < 0.02) and mental health (β 4.20; 95% CI 0.26-8.13; p linear trend < 0.06). As suggested by previous work in this field, these associations were clinically relevant because the β regression coefficients were higher than 3 points. Finally, the number of sitting hours showed a gradual and inverse relation with the scores on most of the SF-36 scales, which was also clinically relevant.

**Conclusions:**

Greater LTPA and less LTSB were independently associated with better long-term HRQoL in older adults.

## Background

Physical activity reduces the risk of numerous diseases, like ischemic heart disease,[1] stroke,[2] diabetes mellitus[3], and cognitive disorders,[4] as well as total mortality [5]. Health-related quality of life (HRQoL) is a global indicator of health resulting from the individual's perception of the impact that diseases exert on different spheres of life (physical, mental and social). Most of the evidence on the relation between leisure-time physical activity (LTPA) and HRQoL has been obtained in cross-sectional studies in middle-age adults [6,7]. However, little evidence exists in the case of the elderly. This evidence is based on clinical trials of the short-term effect of exercise programs in patients with chronic diseases, who are often institutionalized,[8,9] and in cross-sectional studies, which have limited capacity to establish causal relations because HRQoL itself may influence the ability to do physical activity [10-16]. To our knowledge, only one study in elderly women has shown a longitudinal association between higher LTPA and better HRQoL;[17] however, it was limited to examining the effect of LTPA on the mental components of HRQoL, and did not include the physical components.

Finally, there is growing consensus that sedentary behavior has a harmful effect on health independently of the total volume of physical activity performed [18]. Specifically, it is known that a greater number of hours spent sitting down or watching television is associated with a higher risk of cardiovascular disease,[19,20] diabetes,[21] and general mortality [22]. However, we know of no study that has yet examined the influence of the number of sitting hours on HRQoL in older adults. Accordingly, we conducted a longitudinal study of the association of LTPA and number of sitting hours with HRQoL in older adults. This association is important because of several reasons: the elderly are the population segment with the largest increase over the last decades, their HRQoL worsens with age, and LTPA and LTSB are modifiable behaviors.

## Methods

### Study design and subjects

The study methods have been reported elsewhere [23,24]. In 2001 baseline information was obtained from a cohort of 4,000 persons representative of the non-institutionalized population age 60 and over in Spain. Study subjects were selected using probabilistic sampling within multistage clusters. The clusters were stratified by region of residence and size of town. Thereafter, census sections were selected at random in each cluster, followed by individual households where information was obtained from residents. Data were collected on a total of 420 census sections in Spain, with subjects being selected in sex and age strata. Subjects who could not participate after 10 failed visits by the interviewer or because of incapacity, death, institutionalization or refusal were replaced with other individuals selected with the same sampling procedure. Data were collected by home-based personal interview with subjects, followed by a physical examination, performed by trained and certified personnel.

In 2003 the cohort was contacted for the second time with the result that, after excluding the 245 deaths in the first 2 years of follow-up, information was obtained on 2,990 persons by telephone interview. The individuals contacted did not differ significantly from those lost to follow-up in any sociodemographic and life-style related characteristics, except for the number of chronic diseases diagnosed and reported in 2001, which was 1.4 among those remaining in the cohort and 1.2 in those lost to follow-up [24]. Finally, in 2009 study participants were contacted again and, after excluding the 1,105 deaths since 2003, telephone interviews were conducted with 1,608 persons. In comparison with those lost to follow-up between 2003 and 2009, those that were followed up to 2009 were younger, and had higher educational level, lower frequency of sedentary behavior, and fewer chronic diseases.

In this study, LTPA, LTSB and the potential confounders of the study relation, including HRQoL, were measured in 2003, and HRQoL was measured again in 2009. There is evidence in Spain that the validity and reliability of information obtained by telephone interview on lifestyles and HRQoL is similar to that obtained in face-to-face interview [25-27].

Written informed consent was obtained from all study participants and from an accompanying relative. The study was approved by the Clinical Research Ethics Committee of the "La Paz" University Hospital in Madrid, Spain.

### Variables

#### Principal variables

LTPA was evaluated with the Spanish version of the physical activity questionnaire used in the Nurses' Health Study and the Health Professionals' Follow-up Study [28]. This questionnaire rates participation in 16 different activities: walking, dancing, stationary bicycling, bicycling outdoors, competitive running, jogging, gardening, skiing, climbing, football, going to the gym, judo, swimming, tennis, sailing, and other team sports. The time devoted to each activity per week was recorded using nine response categories: 1-4 min/week, 5-19 min/week, 20-59 min/week, 1-1.5 hr/week, 1.5-2.0 hr/week, 2-3 hr/week, 4-6 hr/week, 7-10 hr/week, and more than 10 hr/week. The mean value of each category was used to transform the time devoted to each activity to a continuous variable. The period of the year during which each activity was performed was also determined, using four categories: all year, more than 6 months per year, 3-6 months per year, and less than 3 months per year.

The number of metabolic equivalents (METs) for each activity was calculated using the compendium of Ainsworth et al as a reference [29]. To determine the weekly volume in METs of each LTPA, the number of hours per week devoted to each activity was multiplied by its specific energy expenditure in METs (MET-hr/week). The number of MET-hr/week was weighted by the annual period of participation in each activity. Finally, the total volume of MET-hr/week was calculated as the sum of all MET-hr/week for all activities.

We determined whether individuals met the recommendations of physical activity for older adults elaborated by the American College of Sports Medicine and the American Heart Association[30] (ACSM/AHA), which consist of doing at least moderate activity during ≥ 2.5 hr/week, or vigorous activity during ≥ 1 hr/week. For this purpose, we classified the type of LTPA according to intensity: light (< 3 METs), moderate (3-6 METs) and vigorous (> 6 METs), and participants were grouped into three categories: no LTPA or LTPA intensity of < 3 METs, LTPA intensity of ≥ 3 METs but does not meet the recommendations, and, finally, LTPA intensity of ≥ 3 METs and does meet the recommendations.

Sedentary behavior was estimated by the total number of hours per week spent sitting down, based on the following question referred to leisure time: "About how much time per week do you spend sitting down on weekdays? Please, add up the total number of hours that you spend sitting down for all activities (eating, listening to the radio, watching television, reading, sewing, driving, etc.)." The same question was asked with reference to a weekend day. The number of sitting hours per week was calculated by multiplying the number of sitting hours on a weekday by 5, and adding twice the number of sitting hours on a weekend day.

HRQoL was assessed using the Spanish version of the SF-36 [31]. This questionnaire consists of 36 items grouped into eight scales: physical functioning, physical role, bodily pain, general health, vitality, social functioning, emotional role and mental health. Replies receive a numeric score which is transformed to a scale of 0 to 100, with a higher score corresponding to better health status.

#### Potential confounders

In 2003, information was collected on sociodemographic variables, HRQoL (using the SF-36 questionnaire) and lifestyles such as age, sex, educational level, size of municipality of residence, consumption of tobacco, and consumption of alcohol. Participants were also asked about the following self-reported diseases diagnosed by a physician: coronary disease, stroke, cancer at any site, chronic obstructive pulmonary disease, arterial hypertension and diabetes mellitus. Weight and height were obtained with the following question "Can you tell me about how tall you are and how much you weigh without shoes or clothes?" Good correlation between measured and self-reported weight has been reported in our cohort (Spearman's correlation coefficient = 0.94; p < 0.001) [24]. Body mass index (BMI) was calculated as weight in kg divided by height in meters squared.

### Statistical analysis

Of all the study participants followed, 353 could not be included in the analyses because they lacked important data (347 individuals on the SF-36, and 6 on other variables of interest). Accordingly, the analyses were conducted with 1,097 persons.

To assess the study associations, we constructed three types of linear regression models, where the dependent variable was each of the SF-36 scales in 2009. In the first model, the main independent variables were LTPA and sitting time in 2003, which were introduced simultaneously to evaluate their independent effects. To model LTPA, individuals were classified into sex-specific quartiles of MET-hr/week, with an additional category for those who did no LTPA serving as the reference group. To model the number of sitting hours, subjects were classified into sex-specific quartiles and the lower quartile was used as the reference.

In the second model, the main independent variable was compliance with the ACSM/AHA recommendations in 2003, with individuals classified into three categories: no LTPA or LTPA intensity of < 3 METs, which was the reference category; LTPA intensity of ≥ 3 METs but does not meet the recommendations; and LTPA intensity of ≥ 3 METs and does meet the recommendations. In this model, the number of sitting hours was also introduced simultaneously.

In the third model, we used the approach developed by Mekary *et al*, [32,33] to assess the impact on HRQoL of the isotemporal substitution of LTPA for LTSB. In this model, the regression coefficients estimate the impact on each SF-36 scale of replacing one hour spent seated by one hour performing different types of physical activity.

The three models were adjusted for the main confounders measured in 2003, including sociodemographic variables, HRQoL, lifestyles other than LTPA and LTSB (e.g., alcohol and tobacco consumption), chronic diseases and BMI. All confounders were modeled with dummies, except for age and the baseline value of each SF-36 scale, which were modeled as continuous variables. To test the linear relationship we calculated the p for linear trend by modeling LTPA and sitting time as continuous variables.

Statistical significance was established at two-tailed p < 0.05. The analyses were performed with SAS, version 9.1 for Windows [34].

## Results

The most frequent activities in 2003 were walking (87.7% of subjects), gardening (28.7%), and swimming (19.1%). With regard to total volume of LTPA, the median was 21.3 MET-hr/week, and the 25^th ^and 75^th ^percentiles were 4.6 and 26.3 MET-hr/week, respectively. Men performed more physical activity than women (median MET-hr/week was 23.8 in men and 13.8 in women; p < 0.001).

Table 1 shows the main characteristics of study participants according to LTPA. About 15.7% of study participants did no LTPA. Compared with those who did no LTPA, those in the upper quartile of LTPA were more frequently men, current smokers, consumers of alcohol, had higher educational level, and spent fewer hours sitting. They also had higher (better) scores on all scales of the SF-36.

**Table 1:** Baseline characteristics of study participants according to leisure-time physical activity (LTPA).

		MET hr/week of LTPA					
		
	Total	No LTPA	Quartile 1(lower)	Quartile 2	Quartile 3	Quartile 4 upper)	p value
**Men**, %	40.8	22.7	48.5	53.6	33.2	42.5	< 0.0001
**Age (years) **mean ± SD	70.3 ± 5.6	70.6 ± 5.9	71.3 ± 6.3	70.5 ± 5.7	69.9 ± 5.2	69.3 ± 4.7	0.002
**Municipality of residence**, %							0.003
Rural	50.1	55.3	54.9	45.9	40.9	54.6	
Urban	49.9	44.7	45.1	54.1	59.1	45.4	
**Educational level**, %							0.14
No education	44.7	50.6	44.1	44.2	45.3	40.7	
Primary	39.3	37.8	38.9	37.0	42.1	39.8	
Secondary and university	16.1	11.6	17.0	18.8	12.7	19.5	
**Tobacco consumption**, %							0.002
Never smoker	67.9	79.5	62.3	62.9	70.3	67.2	
Former smoker	21.7	14.9	27.6	22.5	21.9	19.2	
Current smoker	10.4	5.6	10.0	14.6	7.8	13.7	
**Alcohol consumption**, %							0.04
Non drinker	45.1	52.8	44.8	38.4	50.2	39.8	
Former drinker	8.6	9.9	9.6	6.7	8.2	8.5	
Moderate drinker	35.6	30.3	36.7	42.5	29.2	39.2	
Excessive drinker	10.8	7.0	8.9	12.4	12.5	12.5	
**Diseases**, %							
Coronary disease	3.3	5.3	3.8	4.6	2.0	1.6	0.15
Stroke	2.0	1.9	2.3	2.9	1.9	0.8	0.59
Cancer and neoplasms	1.9	1.7	2.6	2.5	1.9	0.8	0.67
COPD	14.9	14.0	15.3	16.3	14.8	13.7	0.95
Diabetes mellitus	18.8	15.2	21.0	23.3	15.1	18.8	0.12
Arterial hypertension	66.2	70.9	68.6	64.1	65.2	62.9	0.41
**BMI (kg/m^2^)**, mean ± SD	29.1 ± 4.3	29.2 ± 4.5	29.2 ± 4.3	29.1 ± 4.9	28.9 ± 3.7	29.1 ± 4.4	0.79
**Sitting hours/week**, mean ± SD	30.9 ± 1.4	31.5 ± 1.4	32.2 ± 1.5	32.9 ± 1.6	29.4 ± 1.4	28.0 ± 1.5	0.02
**SF-36 scales**, mean ± SD							
Physical functioning	74.1 ± 23.4	64.9 ± 25.4	70.3 ± 25.6	74.6 ± 23.2	77.8 ± 20.1	82.1 ± 18.2	< 0.0001
Physical role	74.9 ± 36.6	65.4 ± 40.0	70.5 ± 39.3	78.3 ± 35.5	78.1 ± 34.1	81.5 ± 31.3	< 0.0001
Bodily pain	64.9 ± 29.3	55.8 ± 28.6	62.4 ± 31.5	67.7 ± 29.1	69.8 ± 27.6	68.3 ± 26.7	< 0.0001
General health	58.9 ± 19.4	53.6 ± 19.3	57.7 ± 19.8	57.5 ± 19.4	61.5 ± 18.2	64.4 ± 18.8	< 0.0001
Vitality	65.9 ± 24.5	57.4 ± 24.9	64.7 ± 26.3	64.4 ± 24.9	68.9 ± 22.7	73.7 ± 19.9	< 0.0001
Social functioning	83.8 ± 25.0	74.5 ± 30.4	80.8 ± 26.9	84.0 ± 25.5	89.0 ± 19.2	89.8 ± 18.8	< 0.0001
Emotional role	79.1 ± 35.6	69.3 ± 41.3	77.2 ± 36.3	79.7 ± 36.2	83.6 ± 31.5	85.3 ± 30.1	< 0.0001
Mental health	71.8 ± 22.7	65.0 ± 24.3	71.4 ± 23.0	69.7 ± 24.2	75.5 ± 20.2	77.3 ± 19.5	< 0.0001

BMI: Body mass index; COPD: Chronic obstructive pulmonary disease.

The cut-off points for LTPA quartiles in MET-hr/week were 14.0; 25.0 and 37.5 in men and 10.0; 21.3 and 26.3 in women.

Table 2 shows the multivariate association between LTPA in 2003 and the scores on the eight scales of the SF-36 in 2009. Compared with those who did no LTPA, subjects in the upper quartile of LTPA had better scores on the scales of physical functioning (β 5.65; 95% confidence interval [CI] 1.32-9.98), physical role (β 7.38; 95% CI 0.16-14.93), bodily pain (β 6.92; 95% CI 1.86-11.98), vitality (β 5.09; 95% CI 0.76-9.41), social functioning (β 7.83; 95% CI 2.89-12.75), emotional role (β 8.59; 95% CI 1.97-15.21) and mental health (β 4.20; 95% CI 0.26-8.13). As suggested by previous work in this field, the magnitude of the association was clinically relevant because in all cases the β regression coefficient was greater than 3 points [35,36]. Moreover, a linear trend was observed between LTPA quartiles and all the scales of the SF-36 (p < 0.05), except for general health. Also a certain linear trend was observed for the mental health scale, but it did not reach statistical significance.

**Table 2:** Coefficients (95% Confidence Interval) for the Linear Regression of SF-36 Scales in 2009 on Leisure-Time Physical Activity (LTPA) in 2003.

	**Physical functioning**	**Physical role**	**Bodily pain**	**General health**
	
MET-hr/week of LTPA*				
No LTPA	Ref.	Ref.	Ref.	Ref.
Quartile 1 (lower)	0.85 (-3.46 to 5.18)	1.15 (-6.48 to 8.79)	3.05 (-2.07 to 8.18)	0.79 (-2.23 to 3.82)
Quartile 2	-0.46 (-4.81 to 3.87)	4.22 (-3.46 to 11.91)	0.13 (-5.03 to 5.28)	-0.06 (-3.08 to 2.97)
Quartile 3	6.28 (2.10 to 10.46) ‡	13.11 (5.80 to 20.41) ‡	5.77 (0.83 to 10.71) †	2.54 (-0.35 to 5.45)
Quartile 4 (upper)	5.65 (1.32 to 9.98) ‡	7.38 (0.16 to 14.93) †	6.92 (1.86 to 11.98) ‡	1.48 (-1.52 to 4.49)
p for linear trend	< 0.001	0.001	0.01	0.18
R-square	0.43	0.22	0.32	0.34
	**Vitality**	**Social functioning**	**Emotional role**	**Mental health**
	
MET-hr/week of LTPA*				
No LTPA	Ref.	Ref.	Ref.	Ref.
Quartile 1 (lower)	1.75 (-2.57 to 6.09)	4.60 (-0.34 to 9.55)	4.36 (-2.34 to 11.07)	1.97 (-2.00 to 5.94)
Quartile 2	1.39 (-2.93 to 5.72)	3.28 (-1.69 to 8.26)	4.77 (-1.95 to 11.49)	1.61 (-2.35 to 5.58)
Quartile 3	6.11 (1.95 to 10.27) ‡	9.29 (4.50 to 14.09) ‡	5.19 (-1.24 to 11.62)	2.35 (-1.47 to 6.17)
Quartile 4 (upper)	5.09 (0.76 to 9.41) †	7.83 (2.89 to 12.75) ‡	8.59 (1.97 to 15.21) †	4.20 (0.26 to 8.13) †
p for linear trend	0.004	< 0.001	0.02	0.06
R-square	0.36	0.23	0.11	0.35

* The cut-off points for LTPA quartiles in MET-hr/week were 14.0; 25.0 and 37.5 in men and 10.0; 21.3 and 26.3 in women.

† p < 0.05; ‡ p < 0.01.

Models adjusted for sex (man, woman), age (years), educational level (no education, primary, secondary or university), size of municipality of residence (rural, urban), tobacco consumption (never smoker, former smoker, current smoker), alcohol consumption (non drinker, former drinker, moderate drinker, excessive drinker), coronary disease, stroke, cancer at any site, chronic obstructive pulmonary disease, diabetes mellitus, arterial hypertension, body mass index (quartile 1, quartile 2, quartile 3, quartile 4), sitting hours per week (quartile 1, quartile 2, quartile 3, quartile 4) and score on appropriate SF-36 scale in 2003 (0 to 100 points).

Overall statistical significance (F-test) for each model was p < 0.01.

Study participants may suffer from long-term disability or chronic diseases that limit physical function, so that they have both low LPTA and poor SF-36 outcomes. Thus, for these people, poor SF-36 may have led to lower LPTA. To rule out this mechanism of reverse causation, we rerun the analyses with the 285 individuals who, at baseline, were free of coronary disease, stroke, cancer at any site, chronic obstructive pulmonary disease, diabetes mellitus and arterial hypertension). Results went in the same direction to those in table 2, but the statistical power was necessarily much lower (data not shown). Also to exclude reverse causation, we conducted analyses using as reference the individuals in the second quartile of LTPA (i.e. those showing some minimal capacity to do physical activities at baseline). Results were generally consistent with those in table 2 though, as expected, the association tended to be weaker (data not shown).

About 33.8% of study participants met the ACSM-AHA recommendations on physical activity. In addition, 24.7% of the subjects did not meet the recommendations but performed physical activity with intensity of ≥ 3 METs. Finally, 41.5% did not meet the recommendations either because they did no LTPA or the intensity was < 3 METs. Table 3 shows the multivariate association between compliance with the ACSM-AHA recommendations in 2003 and HRQoL in 2009. In comparison with those who did no LTPA or LTPA intensity < 3 METs), those who met the recommendations had better physical functioning (β 3.93; 95% CI 0.67-7.19), social functioning (β 4.23; 95% CI 0.52-7.93) and emotional role (β 5.50; 95% CI 0.51-10.48). These associations were clinically relevant because β was greater than 3 points [35]. Physical activity of moderate intensity (≥ 3 METs) without meeting the ACSM-AHA recommendations did not show an association with HRQoL on any scale of the SF-36.

**Table 3:** Coefficients (95% Confidence Interval) for the Linear Regression of SF-36 Scales in 2009 on Compliance in 2003 with Recommendations on Leisure-Time Physical Activity (LTPA) from the American College of Sports Medicine and the American Heart Association (ACSM-AHA).

**ACSM-AHA recommendations on LTPA**	**Physical functioning**	**Physical role**	**Bodily pain**	**General health**
	
No LTPA or LTPA intensity < 3 METs (n = 455)	Ref.	Ref.	Ref.	Ref.
LTPA intensity ≥ 3 METs but does not meet recommendations (n = 271)	1.61 (-1.84 to 5.06)	1.14 (-4.95 to 7.25)	1.71 (-2.37 to 5.79)	0.61 (-1.80 to 3.01)
Meets recommendations (n = 371)	3.93 (0.67 to 7.19)*	0.38 (-5.34 to 6.11)	2.70 (-1.12 to 6.53)	-0.13 (-2.42 to 2.14)
p for linear trend	0.01	0.89	0.16	0.92
R-square	0.42	0.21	0.32	0.33
	**Vitality**	**Social functioning**	**Emotional role**	**Mental health**
	
No LTPA or LTPA intensity < 3 METs (n = 455)	Ref.	Ref.	Ref.	Ref.
LTPA intensity ≥ 3 METs but does not meet recommendations (n = 271)	-0.59 (-4.05 to 2.86)	2.12 (-1.82 to 6.07)	4.15 (-1.16 to 9.46)	-0.76 (-3.92 to 2.39)
Meets recommendations (n = 371)	2.39 (-0.86 to 5.65)	4.23 (0.52 to 7.93)*	5.50 (0.51 to 10.48)*	1.10 (-1.85 to 4.07)
p for linear trend	0.15	0.02	0.03	0.48
R-square	0.35	0.22	0.10	0.34

*p < 0,05.

Models adjusted for sex (man, woman), age (years), educational level (no education, primary, secondary or university), size of municipality of residence (rural, urban), tobacco consumption (never smoker, former smoker, current smoker), alcohol consumption (non drinker, former drinker, moderate drinker, excessive drinker), coronary disease, stroke, cancer at any site, chronic obstructive pulmonary disease, diabetes mellitus, arterial hypertension, body mass index (quartile 1, quartile 2, quartile 3, quartile 4), sitting hours per week (quartile 1, quartile 2, quartile 3, quartile 4) and score on appropriate SF-36 scale in 2003 (0 to 100 points).

Overall statistical significance (F-test) for each model was p < 0.01.

Study participants reported sitting for a median of 28 hours per week (the 25th percentile was 21 hours, and the 75th percentile was 42 hours). Median sitting time per week was similar in men and women. Table 4 shows the association between LTSB in 2003, expressed in quartiles of sitting hours per week, and HRQoL in 2009. The results were adjusted for LTPA. In comparison with subjects in the lower quartile of sitting time, those in the upper quartile had worse scores on the scales of physical functioning (β-9.21; 95% CI -13.36 to -5.04), physical role (β-11.96; 95% CI -19.33 to -4.59), bodily pain (β-6.58; 95% CI -11.51 to -1.64), vitality (β-5.04; 95% CI -9.21 to -0.88) and social functioning (β-6.36 95% CI -11.17 to -1.56). The magnitude of these associations was at least as large as those observed for LTPA. The number of sitting hours showed an inverse linear trend (p < 0.05) with the score on all the SF-36 scales except for general health and emotional role.

**Table 4:** Coefficients (95% Confidence Interval) for the Linear Regression of SF-36 Scales in 2009 on Sitting Hours per Week in 2003.

	**Physical functioning**	**Physical role**	**Bodily pain**	**General health**
	
Sitting hours/week*				
Quartile 1 (lower)	Ref.	Ref.	Ref.	Ref.
Quartile 2	-5.99 (-9.79 to -2.20) ‡	-6.84 (-13.57 to -0.11) †	-3.19(-7.70 to 1.32)	-0,38 (-3,05 to 2,28)
Quartile 3	-5.44 (-10.08 to -0.80) †	-4.73 (-12.96 to 3.48)	0.15 (-5.35 to 5.67)	2,46 (-0,78 to 5,72)
Quartile 4 (upper)	-9.21 (-13.36 to -5.04) ‡	-11.96 (-19.33 to -4.59) ‡	-6.58 (11.51 to -1.64) ‡	-2,68 (-5,60 to 0,23)
p for linear trend	< 0.0001	0.005	0.03	0.14
R-square	0.43	0.22	0.32	0.34
	**Vitality**	**Social functioning**	**Emotional role**	**Mental health**
	
Sitting hours/week*				
Quartile 1 (lower)	Ref.	Ref.	Ref.	Ref.
Quartile 2	-0.69 (-4.50 to 3.10)	-2.36 (-6.73 to 1.99)	-2.77 (-8.68 to 3.13)	-1,13 (-4,63 to 2,35)
Quartile 3	-0.68 (-5.31 to 3.96)	-3.11 (-8.43 to 2.21)	-3.03 (-10.24 to 4.19)	-0,67 (-4,94 to 3,59)
Quartile 4 (upper)	-5.04 (-9.21 to -0.88) †	-6.36 (-11.17 to -1.56) ‡	-6.06 (-12.53 to 0.40)	-5,04 (-8,87 to -1,21) †
p for linear trend	0.01	0.008	0.07	0.009
R-square	0.36	0.23	0.11	0.35

* The cut-off points for sitting hours per week were: 21.0; 28.0; 42.0 in men, and 10.0; 21.3; 26.3 in women

† p < 0,05; ‡ p < 0,01.

Model adjusted for sex (man, woman), age (years), educational level (no education, primary, secondary or university), size of municipality of residence (rural, urban), tobacco consumption (never smoker, former smoker, current smoker), alcohol consumption (non drinker, former drinker, moderate drinker, excessive drinker), coronary disease, stroke, cancer at any site, chronic obstructive pulmonary disease, diabetes mellitus, arterial hypertension, body mass index (quartile 1, quartile 2, quartile 3, quartile 4), leisure-time physical activity in MET-hr/week in 2003 (no activity, quartile 1, quartile 2, quartile 3, quartile 4) and score on appropriate SF-36 scale in 2003 (continuous from 0 to 100 points).

Overall statistical significance (F-test) for each model was p < 0.01.

Lastly, table 5 shows the impact of the isotemporal substitution of LTPA for LTSB on HRQoL. Replacing one hour/day spent seated by one hour/day performing light physical activity in 2003 was associated higher scores on the SF-36 scales in 2009. The association was clinically relevant (regression coefficients > 3) and statistically significant (p < 0.05) for the scales of physical functioning, physical role, bodily pain, vitality, social functioning and emotional role. Replacing the same amount of sitting time by moderate or vigorous physical activity was also associated with better physical functioning on the SF-36.

**Table 5:** Coefficients (95% Confidence Interval) for the Linear Regression of the SF-36 Scales in 2009 on the Isotemporal Substitution of Several Types of Physical Activity for Sedentary Behavior in 2003.

	**Physical functioning**	**Physical role**	**Bodily pain**	**General health**
	
Replacing 1 hour/day spent seated by 1 hour/day spent doing:				
Light physical activity	3.41 (0.81 to 6.00) †	10.61 (6.08 to 15.13) ‡	4.22 (1.19 to 7.26) ‡	2.44 (0.66 to 4.23) ‡
Moderate or vigorous physical activity	4.14 (1.92 to 6.37) ‡	1.19 (-2.71 to 5.10)	2.93 (0.31 to 5.56) †	-0.06 (-1.61 to 1.50)
Housework	1.04 (0.25 to 1.85) †	1.68 (0.27 to 3.01) †	1.05 (0.10 to 1.99) †	0.31 (-0.24 to 0.87)
R-square	0.42	0.22	0.32	0.33
	**Vitality**	**Social functioning**	**Emotional role**	**Mental health**
	
Replacing 1 hour/day spent seated by 1 hour/day spent doing:				
Light physical activity	4.14 (1.58 to 6.71) ‡	4.80 (1.84 to 7.77) ‡	4.93 (0.98 to 8.87) †	2.51 (0.17 to 4.86) †
Moderate or vigorous physical activity	2.51 (0.29 to 4.73) †	2.06 (-0.47 to 4.61)	1.03 (-2.40 to 4.46)	0.53 (-1.51 to 2.57)
Housework	0.67 (-0.13 to 1.47)	1.08 (0.14 to 2.00) †	1.21 (-0.03 to 2.45)	0.83 (0.09 to 1.57) †
R-square	0.36	0.22	0.11	0.34

† p < 0.05; ‡ p < 0.01.

Model adjusted for sex (man, woman), age (years), educational level (no education, primary, secondary or university), size of municipality of residence (rural, urban), tobacco consumption (never smoker, former smoker, current smoker), alcohol consumption (non drinker, former drinker, moderate drinker, excessive drinker), coronary disease, stroke, cancer at any site, chronic obstructive pulmonary disease, diabetes mellitus, arterial hypertension, body mass index (quartile 1, quartile 2, quartile 3, quartile 4), score on appropriate SF-36 scale in 2003 (0 to 100 points), number of hours lying or sleeping, and total number of hours spent in all types of physical activity. Sitting hours were not included in the model.

Overall statistical significance (F-test) for each model was p < 0.01.

## Discussion

Our results show that greater LTPA and less LTSB are independently associated with better long-term HRQoL in older adults. This association affects both the physical and mental scales of HRQoL. Specifically, doing more LTPA showed a positive linear trend with physical functioning, physical role, bodily pain, vitality, social functioning, emotional role and mental health. Moreover, meeting the ACSM/AHA recommendations on physical activity was associated with better physical functioning, social functioning and emotional role. Finally, the number of sitting hours showed a gradual and inverse relation with the score on the scales of physical functioning, physical role, bodily pain, vitality, social functioning and mental health.

The literature is heterogeneous regarding physical activity and HRQoL in older adults, partly because of the different study designs used and different ways of measuring physical activity and HRQoL. In a randomized clinical trial with deconditioned institutionalized elderly persons, Dechamps *et al *showed that a tai chi program and a cognition-action program lasting 6 months slowed down the decline in HRQoL, evaluated as limitations in the activities of daily living at 12 months follow-up [8]. In contrast, a non-randomized intervention study with a small sample size in elderly nursing home residents with minor disabilities found no differences in HRQoL associated with a 12-month program of supervised exercise [9]. In the latter study, HRQoL was assessed by limitations in instrumental activities of daily living, tests of cognitive functioning, health tests, and a scale measuring the fear of falling.

Some cross-sectional studies in older adults have shown a positive association between LTPA and HRQoL. Lobo *et al *showed that physical activity measured for 7 days with an accelerometer in older institutionalized adults was associated with better physical functioning, physical role, vitality and less bodily pain on the SF-36 [11]. Likewise, in a study of 112 healthy volunteers aged 60 and over, more physical activity was associated with better score on various scales of the SF-36 [13]. In addition, in the Behavioral Risk Factor Surveillance System survey in the United States, the proportion of persons age 65 and over who reported 14 or more unhealthy days (physical or mental) was lower in those who did moderate or vigorous physical activity than in those who did not [14]. Finally, in a small Japanese study, in persons aged 65-85 years with physical activity measured by accelerometer for one year, HRQoL was poorer among those in the lowest quartiles of both step count and duration of activity > 3 METs [16].

In one longitudinal study in older adults, women who maintained or increased their physical activity improved their scores on various mental health scales of the SF-36 with respect to those who were always sedentary [17]. Our study extends knowledge on the longitudinal relation between LTPA and HRQoL because it includes standard measures of all the dimensions of HRQoL. Furthermore, our study is unique in showing an inverse association between number of sitting hours and HRQoL in the elderly, which is independent of the total volume of LTPA.

Because the total amount of leisure time is finite, a reduction in LTSB requires the increase in the time devoted to several types of physical activities, which can be heterogeneous in terms of intensity (light, moderate, vigorous) but also in terms of the relative expense and sacrifice needed to engage in them. As pointed out by Mekary et al,[32] the advantage of the isotemporal model is that it allows comparing substitution of a fixed time of an activity type for the same time engaged in sedentary behavior; it, thus, helps to answer the relevant public health question of how to spend the leisure time for optimal HRQoL. Our results suggest that many dimensions of HRQoL can be effectively improved by the isotemporal replacement of LTSB by physical activity of just light intensity (e.g., walking, dancing, gardening); moreover, in our population, replacement of LTSB by activities of moderate-to-vigorous intensity led to better physical functioning but not to clinically relevant improvements in other dimensions of HRQoL.

The enhanced HRQoL associated with increased LTPA may be a consequence of several mechanisms, such as reduced cardiovascular risk factors, prevention and management of chronic diseases,[37,38] lower risk of falls,[39] prevention of functional limitation[40] and lower risk of mental disorders like depression and anxiety[41] and cognitive decline [42]. Another possible mechanism is satisfaction arising from self-efficacy for physical activity. McAuley *et al *suggest that physical activity directly influences self-efficacy and, through it, acts on HRQoL, especially on the components of mental health [15,43,44]. Our results are consistent with these studies, since improvements were observed on the mental scales from the first quartile of LTPA, whereas for the scales of physical functioning and physical role clinically relevant effects are not seen until the third quartile (table 2). Longer sitting time has been associated with overweight and obesity, independently of physical activity [45]. Obesity, diabetes and hypertension are possible mediating mechanisms that may explain the association between sedentary behavior and HRQoL [46]. Furthermore, most sedentary activities, such as watching television, reading or sitting at the computer, decrease communication with the family, reduce the social network[47] and increase the risk of depression, anxiety and stress,[48] which would explain the poorer quality of life associated with sedentary behavior.

Our study has several strengths and limitations. An important strength is that the we used validated instrument to measure LTPA and LTSB [28,49] as well as HRQoL [31,50]. Another strength is that our analyses were adjusted simultaneously for LTPA and LTSB, and for a considerable number of confounding factors.

Losses to follow-up were the main study limitation. Measuring HRQoL in older adults over the long term is complex, since the SF-36 does not allow for replies by third parties, and it is inevitable that a certain proportion of elderly persons experience mental and/or physical decline that impede completing the questionnaire over time. Losses to follow-up could affect the representativeness of our cohort; nonetheless, the association between LTPA and HRQoL had already been observed in a cross-sectional analysis of this cohort in 2001 [23]. Another study limitation is that the question on sitting time does not allow to know the specific activity done while seated (watching television, reading, driving, etc.); thus this study cannot make specific recommendations to reduce sedentary behavior. Lastly, studies on the effect of physical activity on health are potentially affected by a reverse causation bias, because individuals with better health status and less disability are more able to do physical activity. However, the analyses conducted with participants free of disease at baseline, and those using the second quartile of physical activity as reference, produced results in the same direction to those in the whole study sample. Although it does not entirely rule out reverse causation, it suggests that its contribution to our results is likely to be small.

## Conclusions

Greater LTPA and less LTSB were independently associated with better long-term HRQoL in older adults. These findings have practical importance because they illustrate that LTPA can reduce the age-associated decline in HRQoL. Moreover, most of the LTPA in our cohort consisted of walking, which is the safest activity in older adults,[51] and there is evidence of the short-term efficacy of brief counseling and the use of pedometers to increase the time spent walking [52]. Finally, this study suggests that HRQoL might be improved by replacing time spent seated by time performing light physical activity, and that doing it for just one hour/day may have clinically relevant benefits.

## List of abbreviations

**HRQoL: **Health-Related Quality of Life; **LTPA: **Leisure-Time Physical Activity; **LTSB: **Leisure-Time Sedentary Behavior; **METs: **Metabolic Equivalents; **ACSM/AHA: **The American College of Sports Medicine and the American Heart Association

## Competing interests

This study was partially funded by FIS grants PI08-0166 and PI09-1626. Authors have no financial or any other kind of personal conflicts with regard to this paper.

## Authors' contributions

TBC, LLM, AG, FRA and PGC contributed to study concept and design, analysis and interpretation of data. TBC, FRA and PGC drafted the manuscript. FRA and PGC provided study supervision. All authors reviewed the manuscript for important intellectual content and approved the final version.
